# Arsenious Acid in Apoplectic Congestions

**Published:** 1860-03

**Authors:** 

**Affiliations:** The Hospital of Honfleur


*Arsenious Acid in Apoplectic Congestions.* By Dr. Lamarc-Picquet, of the Hospital of Honfleur.

The author gives, as the result of his investigations on this subject, the following conclusions: Apoplexy is essentially misunderstood, since the effusion of blood is only a secondary phenomenon. It is

* The translator must dissent from the statement of the author here. The voice of the profession is demanding that ether be the sole agent employed in producing anæsthesia.

easy to master the preliminary symptoms of apoplexy, which is owing to an undue increase of blood-globules. Arsenious acid is a valuable therapeutic resource in all congestions of the cerebral apoplectic form, since its first effect is to render the blood less rich in globules and less plastic. It is, however, indispensable, before the use of the agent, that the richness or condition of the blood should be determined, as, in case this fluid be poor in globules, the use of arsenious acid would increase such an abnormal condition.

The action of arsenious acid is connected so intimately with the digestive process, that it should be employed while one is at the table, in order to facilitate its assimilation. The agent should also be used for some time after the cure is effected, in order to increase the probability of the duration of the cure. The agent cannot be considered, however, absolutely antidotal; and hence the physician must always consider the mode of living, idiosyncrasies, and pathological condition of his patient. The dose is usually from four milligrammes to one centigramme (from $\frac{1}{16}$ to $\frac{1}{6}$ of a grain) a day.

The author employed this treatment in his own case. He had been laboring under all the annoying symptoms which seem to predict an apoplectic attack—sense of weight in the head, and feeling of constriction. His age was then fifty-six, constitution robust, temperament very decidedly sanguine. From October 29, 1845, to March, 1849, he treated himself by bleeding, bicarbonate of soda, and other agencies, but at last, having perceived the effects of arsenious acid, he determined to try it in his own case.

"March 23, 1849, I commenced the arsenical treatment, employing five milligrammes, at breakfast and dinner, in water, and used a vegetable diet most strictly, except when very much fatigued, when fresh fish was employed. This dose of the medicament was employed for a month without the least inconvenience to the digestive process. May 3, I was perfectly well, although fifty days had passed without the use of bloodletting. A small quantity of blood being drawn on that day, it furnished fifty-two parts of cruor to the hundred. (In previous bleedings, the per centage ranged from 58 to 75.) Cerebral sedation had been completely gained, and the elements of the blood were in normal proportions. It was necessary to preserve this state of affairs, and I continued the use of the agent. The dose was even made as large as sixteen milligrammes. Occasionally the medicine was suspended for eight or ten days, then resumed for a period of fifteen or twenty days. This treatment continued until November, 1849. Then, I experienced some gastric troubles, (weakness of stomach and flatulence;) and I ceased to take the arsenical solution, when the nervous symptoms disappeared. Returning occasionally to the use of the medicine, in order to steady its action on the stomach, the same nervous phenomena always showed themselves—yielding to its discontinuance. The dose was reduced to eight milligrammes a day, which never produced any gastric derangement."

"After November, 1849, my usual mode of living was adopted, as before the year 1845. I could eat game, and even take a glass of wine, which I had not done for more than four years. Up to the month of March, 1850, no symptom of cerebral excitation was experienced, but then I experienced some of those flying symptoms which always indicated the approach of a congestive condition. On being bled, with a view to examine the composition of the blood, found that there was 58 per cent. of cruor. The arsenical solution was again taken, four milligrammes morning and evening; and this treatment was continued until May, without returning to a vegetable diet; making my meals of meat, legumes, and fruit, and using water as the only beverage. During the month of July, 1850, I enjoyed the best possible health, the heat of the summer and a more substantial diet producing no unfavorable effects."

"From that time, for nearly five years, no arsenious acid was taken, and no cerebral trouble was experienced. On the night of January 5, 1856, however, I was troubled with a nightmare, which wakened me with a start, and left me laboring under a species of noisy sounds in the head, continuing until morning. During the following days, I experienced a sensation similar to that produced by a cap tightly drawn around the head, and the continuance of this symptom brought to mind my previous attack. Venesection was performed, to the amount of fifteen or twenty grammes, so as to get an idea of the nature of the blood; the per centage of cruor being 66. I resumed the use of arsenious acid, in dose of ten milligrammes, and continued it until March, when the quantity was reduced to eight milligrammes. In May, being perfectly well, the treatment was discontinued. I am now in my sixty-sixth year, and age has of course not diminished the predisposition to apoplectic congestions. In March, 1859, I suffered from ringing sounds in my ears and pains in the head, (the countenance was more colored than usual,) awkwardness in, and fatigue from, locomotion, disturbed and troubled sleep. These symptoms induced me to examine the condition of the blood, which showed 64 per cent. of cruor. I immediately resorted to arsenious acid, in dose of six milligrammes, and I still enjoy the beneficial results of the treatment—my head is perfectly clear, and my body and spirits are active."

This last fact furnishes me the following bit of instruction:

In subjects constitutionally sanguine, morbid predispositions, depending on that condition, such as cerebral congestions, can be modified for a longer or shorter time, but nature tends always to assume its rights. It becomes a man, in the decline of life and in old age, when his mind is not injured, to hearken to those preliminary indications that announce its destruction. As a corollary to what I have written, it may be said, that arsenious acid cannot always modify apoplectic predispositions, but that, in the slightest congestive tendency to the brain, persons of a plethoric habit should have recourse to an agent which will promptly check this congestion, and continue its use long enough to prevent a relapse.—*Bull. Gén. de Thérapeut., Sept.*, 1859. L. H. S.

